# The History, Diagnosis, and Treatment of the Fevers of the United States

**Published:** 1857-03

**Authors:** 


### THE NORTH AMERICAN MEDICO-CHIRURGICAL REVIEW.

MARCH, 1857.

### Analytical and Critical Reviews.

ART. I.—*The History, Diagnosis, and Treatment of the Fevers of the United States.* By ELISHA BARTLETT, M.D., late Professor of Materia Medica and Medical Jurisprudence in the College of Physicians and Surgeons of the University of the State of New York, &c. &c. Fourth Edition, Revised by A. CLARK, M.D., Professor of Pathology and Practical Medicine in the College of Physicians and Surgeons of the University of the State of New York. Philadelphia, 1856. Blanchard & Lea. Pp. 610, 8vo.

*Diseases of the Circulatory System.* Division,—Fevers,—from p. 173 to p. 337, in Part II, of "Elements of Medicine: a Compendious View of Pathology and Therapeutics; or the History and Treatment of Diseases. By SAMUEL HENRY DICKSON, M.D., LL.D., Professor of the Institutes and Practice of Physic in the Medical College of the State of South Carolina." Philadelphia. Blanchard & Lea, 1855. Pp. 752, 8vo.

MANKIND have ever been fond of personifying death with dart raised, and ready at every moment to strike the victims who are continually falling beneath his attacks. But indiscriminate as these may, at first sight, seem to be, we find on observation, that particular classes suffer more than others; and that they are ticketed for their doom by certain very distinctive marks, recognized by the term disease, of which the number is great and the physiognomy correspondingly varied. Of the diseases which announce the doom of death's victims, none are of such sinister augury, and usher so many out of the world as Fevers. None have interposed such serious obstacles to the schemes of ambition, whether directed to conquest, or to commercial aggrandizement and the acquisition of wealth in the great marts of trade. The wisest combinations of genius by the general, and the tried valor of his army, are often set at nought by febrile pestilence. It can be shown, that it is more destructive in the end, than that "infernal fire," which compelled at last the Russian commander to yield Sebastopol to the allied French and English forces. Much as these latter suffered in the trenches, and in successive sorties, and great as was their loss in the bloody fields of Alma, Inkermann, and Balaklava, they encountered more protracted suffering and greater losses from fevers. So it has been from the earliest times to the present; and so, without a change in nature and unlooked-for revelations in medical science, will it continue to be in after times. The chosen sites for the emporiums of commerce are, for the most part, at the mouths of rivers, or at distances easily reached from the ocean, where the land is low and marshy, and, as such, subjects the inhabitants to one kind of fever,—periodical, which, with increasing population, and crowding, and scant space for lodging, is replaced often by another kind, or the continued. Colonial settlement and the clearing of the forest for the wants of agriculture, are also followed by fevers, which are, in fact, the sorest obstruction in the path of the adventurous emigrant, even when, as is too often the case, his progress is not entirely arrested by death from their attacks. Mournful distinction has been gained for some countries and districts by the excessive mortality from fever, which had assailed armies during campaigns in them, or expeditions engaged in their capture. Thus, for example, we read of the Hungarian fever, the Walcheren fever, and the Batavian fever, and, on our own continent, the Mexican fever; all of which have committed from three to five fold the havoc of lives, and even some of them a still greater proportion, beyond what were lost in battle or in siege. Wherever our fellow-creatures are engaged in war, we find them in large numbers, assailed to the death by these great destroyers, which do not cease their ravages when men are engaged in the pursuits of gainful commerce and manufactures, or in the more tranquil and less risking ones of agriculture.

On a theme like this, in which every one may be said to have a lively personal interest, depending either on actual or prospective experience, there can be no lack of description nor backwardness in descanting on its portentous features. There is, unfortunately, another reason for the subject of fever continually inciting us to inquiries and to speculations likewise, viz., in the circumstances of the mystery in which its causes are shrouded, and of the difficulties which still embarrass us in all our attempts to arrest its progress, either in its endemial or individual manifestations. Every now and then, we are tantalized by the exclamation of some enthusiast, who cries out "*Eureka*," I have found it, meaning sometimes a knowledge of the remote cause, sometimes of its treatment; but sober experience comes along, and the sounds of boastful promises are hushed; proofs fail, the evidence is insufficient, and each inquirer is left to continue his observations, and to pursue his quest after the concealed treasure, in company, perhaps, with others who are bent on discovering the quadrature of the circle, perpetual motion, or the elixir of life. But, short of the extremes of what may be called the first causes and the specific treatment of fever, there is a wide field for legitimate investigation, in which enough of the certain has been found to reward the labor of search, while there yet remains a sufficient share of the doubtful and the unknown to stimulate us continually to renewed exertions to reach farther discoveries. In this respect, the search into the characteristics of fever, and the means of staying its ravages, is like the search after a Northwest Passage, or the successive attempts at exploration in the interior of Africa; both in the repeated failures to solve the problem, and in the self-sacrificing and devoting spirit by which they have been inspired, who gave themselves up to the task.

Much has been said of the advantages of mathematical studies in disciplining the mind, by imparting to it habits of patient observation and continuous attention, with exacting methods of induction from the previously ascertained facts or phenomena, whether these be represented by quantities, by properties, or by both. To the student of medicine better mental exercise can hardly be found, than in the study of fever, which demands, together with a rigid scrutiny of carefully-observed phenomena or symptoms, a specification of each, and the order of sequence in which they appear, constituting a numerical analysis. This must be followed by a description of the union of symptoms in groups, and of their alternations, which cannot, any more than the precise and uniform order of the subsidence or disappearance of each of these phenomena which, collectively, make up the diagnosis of fever, be given in numbers. Each symptom represents some physical change; but the mode of succession and alternation of symptoms, and the rapidity with which these are effected, are not to be measured by numbers; nor are they reducible to any known physical laws: they are as incapable of methodical reduction to a fixed value, or representation by set formulæ, as would be the numbers and forms in a geometrical problem when thrown out of their proper sequence and connection, by a sudden and apparently capricious transposition. Limits are soon put to the most indefatigable observations of the phenomena of fever, and when the mind has exercised for the purpose its perceptive faculties, it calls into play those of reasoning and deduction: but finding a certain hiatus which cannot be supplied to make out an unbroken order of sequence, and to allow of a positive induction, it is prone to pass from physics into metaphysics—and without always acknowledging to itself the transition from the inductive to the ratiocinative. One great difficulty in the whole matter consists in a too great readiness to give the perceptive faculties rest before they have discharged their duties to the uttermost, by a patient observation of all that can possibly be observed; another is found in their prematurely invoking, while they are engaged in their duty, the assistance of the reasoning faculties, so as to anticipate conclusions from evidence yet imperfect. A third difficulty lies in receiving as explanations in chief, concurrent phenomena furnished by mathematics and chemistry, and admitting them as causes, when they are merely correlative effects of a general and remote cause not yet ascertained. Instead of trusting to other sciences for a solution of the problem, all the materials of which are not embraced by them, the student of fevers, as indeed the student of medicine entire, ought to strengthen himself by sound logic, so that after having collected all the data cognizable by the senses and perceptive faculties, he may be able by its aid to detect what relations must subsist between data and whatever can be concluded from them, between proof and everything which it proves. Logic cannot give us proofs, but it teaches us what makes them proofs, and how we are to judge of them. In the study of fevers we must enforce a logic, which includes the operation of naming, and also definition and classification.

Considerations of this nature come up in our minds, when looking through the works whose titles are at the head of the present article. They are the productions of men of mark, writers and teachers of medicine, and, at the same time (we wish we could always add as a necessary sequence), scholars of matured judgment and chastened taste; good representatives, also, of the medical literature and style of composition of two great divisions of our country,—the North and the South. Dr. Bartlett's volume treats *ex professo* of the fevers of the United States. Dr. Dickson's embraces a description of fevers more comprehensive, but in less detail, in his "Elements of Medicine." Of the able manner in which the former has treated his subject, stronger proof can hardly be adduced than the fact of the present being the fourth edition. Equal time being allowed, it requires no gift of prophecy to predict a like success for the latter.

It was not allowed to Dr. Bartlett to superintend this edition of his work. Death, which so often, sternly and with seeming caprice, interposes to prevent a continuation of labors for the good of humanity, arrested the author in his onward progress. In the language of his editor and friend,

Dr. A. Clark, who has acted on the present occasion as his literary executor with equal discernment and affection: "The distinguished author of this volume had performed his last scientific labor when his third edition was prepared for the press. Sixteen months ago [from November, 1856], he closed his brilliant professional career, after years of growing bodily weakness and pain; his mind not dimmed by his physical infirmities, but bright and comprehensive, glowing with the memories of the past and the visions of the future. He died too soon for the profession he adorned." For ourselves, we would not curtail, by a single word, the lines of eulogy which follow in the preface; and we would join his surviving friends and admirers in planting "forget-me-not" on his grave, and help, on a fitting occasion, to weave a chaplet of laurel to wreathe the brows of a commemorative bust. But, just now, our notice will not be of the dead author, but of the living work; and what we have to say will be meant for the benefit of the living of those for whom it was written, and whom he hoped to instruct through its pages.

The first edition of Dr. Bartlett's book on fevers was restricted to the history of typhoid and typhus fever. In the second edition he introduced the history of periodical and yellow fever, "thus rendering," to use his own words, "the work what it professes to be, a Systematic and Methodical Treatise on the Fevers of the United States." The proportion of pages taken up with the original subjects in the present volume is nearly three-fifths of the whole number. Some ambiguity is often unavoidable in the common titles of works. In the instance before us it might be asked, whether we are to understand it to mean the history of fevers peculiar to the United States, or of fevers of general and common occurrence in this country, which have been so long connected with its climate, geography, and the circumstances in which its people are placed, as to make them truly national, if not indigenous. In receiving this as the meaning which the author intended to convey by his title, we are arrested by a difficulty and apparent contradiction, when he assures us, at p. 334, "that *typhus fever*, without lesion of the intestines, is a form of disease rarely met with in this country, except among emigrants recently arrived from Europe; and that no clear and well-defined difference is recognized between this and the form of continued fever generally prevalent here." Notwithstanding this, the fever called typhus, separated, in the opinion of the author, from typhoid fever, mainly by the absence of the lesion just mentioned, is made the theme of the second part, amounting to twelve chapters, of the work before us, although, as in no small degree a recent importation, it is scarcely entitled to a place in the history of American fevers; certainly not more than the newly-arrived emigrants, who bring it with them, would be to notice in the ethno-political history of the people of the United States. But, be this as it may, the author could not well have omitted a tolerably full notice of typhus fever, mixed up as the subject is with his ably-written augmentative history of typhoid fever.

He tells us in his Preface, "I thought that the wants of medical science here, at least, demanded a history and comparison of the two chief forms of continued fever, as they are now ascertained to exist, fuller and more discriminating than had yet been written; and these wants I have endeavored to supply." The two chief forms of continued fever adverted to are typhus and typhoid. And again, he declares that one of his "leading purposes has been to bring out more clearly and strongly than has hitherto been done, our means of diagnosis between the different species or forms of fever, and to ascertain and establish their nosological relations." In his history, he describes but four individual forms of fever, "to wit, typhoid fever, typhus fever, periodical fever, in its three forms of intermittent, bilious remittent, and congestive, and yellow fever." The simple reason for this is, that he does "not know anything of any other distinct fever amongst us." In the execution of the several divisions of his history corresponding with those of fevers just made, considerable inequalities are observable. Those on typhoid and typhus, especially the former, both in the "part" formally allotted to it, and in that in which the latter is the chief theme, are treated with the most fulness and augmentative ability. The third "part," on periodical fever, is wanting in detail and completeness. We are not required to speculate on the causes of those inequalities, for the author, in assigning the reason for his beginning his history of the fevers of the United States with that of typhoid, tells us with becoming frankness, that his "own knowledge of the disease derived from personal observation is much more extensive than of the other forms of fever."

Dr. Bartlett gives one of the articles on preliminary matters, which constitute his first chapter, to the names of the disease. He adopts "the term *typhoid fever* as the name of this disease, simply because it is not particularly objectionable, and because it seems to be coming into general use." Here, at the very outset, we are at issue with the author, and we have no hesitation in considering the expletive typhoid, when applied to the fever in question, as particularly objectionable in any writer who, in the general argument, takes the side of the essential difference between typhoid and typhus fevers. He ought to do his best, if not to expunge, at least to withdraw it. If held to as a designating and distinctive title, it must damage beyond retrievement the side taken by the author. It cannot, as such, retain its place so long as typhus is admitted with all the circumstances of symptomatology with which it is universally associated, nor so long as logic, which regulates the selection of names, and the existing analogies of the language are allowed their proper weight. We make the disclaimer thus early, because of his early annunciation of what we believe to be a term in contradiction with the creed of the author; but we shall not enforce our opinion until we have reached a more advanced stage of the general argument, and brought out the features of the two forms of fever on the same canvass. We shall then be able, we think, readily to justify the position which we now take in the premises. On the other hand, if we admit, with so many good observers and acute reasoners, that typhoid is only a modification, a less violent form of typhus fever, then nothing can be more apposite than the use of the common term.

The author, in the "article" devoted to the history of typhoid fever, has, it seems to us, restricted himself within too brief a period, when he dates its beginning from the first description of its pathological lesions by Prost in 1804. We would be among the last to undervalue the importance of the part of the anatomical history of any one fever, or of fevers in general; but its absence by no means implies that a history, and a very useful one too, has not been written. Unless this be conceded, the task of retrospective study will be very light, and we shall have no occasion to inquire into the views, or be influenced by the precepts, of Hippocrates and Sydenham, and many others of our instructors, if not guides, in much of what relates to fevers. Were it to be alleged that the author did not deem it expedient to engage in retrospection beyond the time when the fever received its present name, and had its distinctive characters pointed out, we would reply, that he must then abbreviate still more his historic period, and waive the accounts of the anatomical lesions described by Prost, as well as by Petit and Serres, and by Rœderer and Wagler, and by Bretonneau; as these lesions were noted in a fever which did not receive from these writers the name of typhoid. By some observers it was called *mucous*, by some *entero-mesenteric fever*, by others *dothinenterite*, and by a fourth party *follicular enteritis*. It is, therefore, by constructive, not direct, testimony that he adduces the observations of the above writers as of those "who studied particularly the intestinal lesions of the disease." Conceding that the author was right in his construction of the evidence resting on intestinal lesions being applicable to the cause which he advocates, and that it enters appropriately into his history, we cannot see on what ground he should reject the histories of an earlier date, in which descriptions of a fever under another name, are so clearly written as to admit of no dispute respecting its identity with that now called typhoid. We may specify, for example, Huxham's chapter on the *Slow Nervous Fever*, "which," to use the language of the historian himself, "hath been very exactly taken from too many, who have fallen victims to this insidious and dangerous enemy." In another part of his work (p. 271), Dr. Bartlett tells us that, by some of the older

British physicians, amongst whom may be mentioned especially the incomparable Huxham, the difference between the two forms of fever (typhoid and typhus) was distinctly noticed. So, also, we read, at p. 96, that Hillary, in his account of the diseases of Barbadoes, describes a slow nervous fever, which was very evidently typhoid.* We are, therefore, free to express a wish that the author had given a wider retrospective range to his history of typhoid fever—in justice to the literature, and for a fuller comprehension of the subject.

A reason for the curtailment now adverted to may probably be found in the next article, on "Methods of Description," in which the author presents the two that may be adopted in describing a disease. The one, the most ancient, which may be called the physiognomical, consists in a portraiture or "a general enumeration of the more striking and obvious phenomena of the disease," and which enables us to see at a glance its form, outlines, and features. "The other, which has been followed by many writers within the last fifteen years, especially amongst the French, consists, not merely in this general enumeration of the phenomena, their combinations and progress, but in a thorough and careful analysis of these phenomena; in a special and particular study of each individual element which goes to make up the disease, and in a strict estimate of the relative value and importance of each and all of these several elements." Although the author admits, in the one of these methods, the absence of that wholeness and unity of impression which are made by the other, he declares his preference for the last-mentioned, or the analytical process, as the only one capable of enabling him to point out "the characteristic features of each of the four great forms of idiopathic fever; to establish, as far as possible, a clear and positive diagnosis; to ascertain the resemblances and the differences between them." Here we have a distinct declaration of the manner in which the author intends to write his history of the fevers of the United States. Impliedly, he rejects the propriety or advantage of other fashions of historical writing, both by this his declaration of his own plan, and by his omitting from the list of historians of the fever which he calls typhoid, those who have not pursued the analytical or numerical method of description—as, for instance, Huxham and Hillary. That the physiognomical method of description has its value is, however, practically acknowledged by Dr. Bartlett, in the readiness with which he and others are able to see at once, in the histories of the authors just named, the same features which distinguish the typhoid fever of the present day—even although no mention was made of intestinal lesions.

Enumeration, by furnishing details, helps us to description, and chronicles furnish the primary materials for history; but to enumerate is not to describe,

* In the text, by a typographical mistake, Minorca is put for Barbadoes.

nor does the writing of chronicles constitute history. We may enumerate all the features of the human face; giving the length of the nose and the chin, the prominence and breadth of the cheek-bones, the size of the mouth and of the eyes, the color of the eyes and of the eyebrows and eyelashes, and the extent of surface of the forehead, and whether it is erect or retreating, and yet still fail to convey a picture of the recognizable expression of the countenance. We may chronicle a series of events in the order in which they transpired, and give the names of the chief actors, and still only represent the skeleton of history, which requires to be clothed with flesh, and animated with the nerves and senses, corresponding with a description of the countries and the localities which were the scenes of action, of the character of the people, their social and business habits and tastes, the disposition and intelligence of their rulers, and their motives for taking part in the events narrated. The more we reduce anything to its elements, the less value, in reference to the whole, has each separate element, and the more complete often is the change or loss of the original qualities by which it was distinguished from other things. To say, for example, that a certain substance has a given percentage of carbon, imparts a very faint, if any idea of the class of bodies to which this substance belongs; nor can we, after its complete analysis, which is at the same time destructive, predicate of the reunion of the elements, a substance identical with the first, or of a change in the proportion of the elements, the kind of body, or the properties which it will manifest, by their combination. Analysis, in these instances, gives us a knowledge of constituent elements and of quantity, but yields no explanation of certain sensible properties, as of sweetness, or bitterness, or of the different degrees of these properties. How much more difficult must the task be to analyze or resolve into elements which should represent a fixed value, not of a thing or substance, but of a state or condition of things, such as vital phenomena and disease, which are continually changing, and of which at best we can only make a picture, but without being able to measure entire by a mathematical standard, or determine the recurrence and alternation of each separate phenomenon, or symptom, by numeration. So far as this last can be carried out it is useful by giving us points of comparison of the symptoms and lesions in two different fevers or other diseases; but we must never forget that it is a comparison of certain elements of two diseases, but not of the diseases themselves, which are composed and complex, and that the field of observation and of deduction is different in the two cases. We may note the appearance, after death, of a straw-colored liver in yellow fever, of a bronze-colored liver in remittent fever, and of intestinal lesions in typhoid fever; but such appearances do not, as far as we can learn, contribute to the diagnostic or characteristic features of these fevers respectively, which belong to a state of life. Nor have these lesions any significance even in empirical therapeutics, or such as might be enlisted to obviate the more prominent and distressing symptoms.

If we assume that the symptomatology of fever results from internal changes and lesions, still no one symptom, however strong and marked, is distinctly diagnostic; but its value is relative, and depends on its connection with other symptoms. In a particular stage of typhoid fever, for example, a certain condition of the tongue, dry, with a brownish coat, and crusted or in fissures, so as to bleed when efforts are made to clear the mouth, is observed; yet we have seen precisely this kind of tongue exhibited every morning for years in a person who, though not in good health, was able to discharge the daily duties of his vocation. So of a frequent pulse and *subsultus*. Each separate symptom may have been noted until the list becomes quite large, but still their enumeration fails to convey an idea of the actual appearance of the patient under disease, or, in other words, of the physiognomy of the disease, which is the collection, combination, union, and sometimes fusion of the symptoms, forming an entire picture, not reducible to or explicable by lines or numbers. The difference in the physiognomical expression of two individuals is admitted to depend on certain differences in the features of the face; but these differences cannot be measured by rule and compass, nor are they susceptible of numeration. A painter makes each part of the face, mouth, nose, eyes, &c., a subject of preparatory study and drawing, and he may have acquired the art of painting a mouth, or a nose, or an eye, with great accuracy and beauty; but years of practice in this way will not enable the artist to paint these several features in their relative position and junction with each other, nor even to appreciate the full expression which each is capable of conveying, until he portrays the whole face. Nor must he stop here, but take the portraits of a number of individuals who, severally, manifest a different physiognomy. He soon discovers that the expression of the countenance and the play of features, which tell of the state of the feelings, are not dependent on the size, prominence, or fulness of the several organs or constituent parts of the face,—the nose, lips, cheeks, and eyes—but rather on the contraction under nervous influence of minute muscles, represented by slight touches of his brush, which connect the angles of the mouth and the nostrils with the cheek, but without actually belonging to either, and others which draw together the eyebrows and contract the skin of the forehead. These are vital movements which, together with the beaming eye, are the soul of expression: they are compound results, not reducible to number or measure, nor to be described by an anatomical analysis of the bones, and muscles, and nerves concerned in these movements. The physiognomy of disease, the altered expression of the face under the pain and derangements caused by fever or other acute diseases is as incapable of analysis by a simple enumeration of the color of the skin, and the suffusion and injection of each part separately, as would be the strong passionate emotion of an individual by a description of the size and appearance of each feature separately. It is the *calida junctura*, the entire and active union and blending of the features, which both the physiognomical painter and the diagnosticating physician must study and acquire a familiarity with. It is a knowledge of this kind gained by an experienced nurse, which often enables her to tell the pathognomonic symptoms, and prognosticate the termination of a disease, without her being able to convey by words, or by any numerical or other analysis, the reasons for her judgment.

In thus commenting on Dr. Bartlett's choice of a method of describing disease, we do not mean to impugn its value in so far as it directs us to the study of each individual element; but we are decidedly of opinion that, even when carried out with all possible minuteness, the task of description is only half done. The other half consists in representing the combination of these several elements, the reconstruction of the entire physiognomy by the reunion of its several parts—the synthesis as well as the analysis. More correctly speaking, the actual appearances—the external features, and posture, and the symptoms of internal disturbances,—should all be presented in their totality, and in the connection and in the order in which they actually occur, before the work of separation, the taking to pieces, the analysis is attempted. The author was able, at once, to recognize in the writings of Huxham and Hillary, his typhoid fever, although described by them under another name. We do not believe that it will be equally easy for a succeeding writer to establish with the same readiness the kindred relations of typhoid to another fever, the precise nature of which it is desired to determine at the time. We are sure that it would be a very difficult thing, if it could be done at all, for a student or other person, who had never seen typhoid fever, to recognize its features in the analytical description of it given in the volume before us. The same remark applies to the method pursued in describing, we may rather say in enumerating, the several symptoms of the other fevers,—typhus, periodical, and yellow.

Thus far, we have dealt with the principles, the fundamental positions, laid down by the author for his government in writing his history. These, we have ventured to intimate, are not adequately comprehensive; but all must, we think, acknowledge, that, within the limits which he has prescribed to himself, he has carried out the divisions of his subject with due method, and given the details with desirable precision, and in a style at once clear and expressive. We are not called upon to follow him in his divisions and details; and shall only make use of them so far as may be necessary to elucidate the great question of the actual relations to each other of typhoid and typhus fevers, on which he argues well and plausibly, if not convincingly, with the design of proving that they are essentially and fundamentally different and distinct diseases. If he has left the question still unsettled, his want of success must be attributed to the difficulties with which it is beset, rather than to any want of industry, ingenuity, or honesty of purpose on his part.

The actual relations, whether of close affinity bordering on identity, or of difference marked by broad contrasts, between typhoid fever and typhus fever, can only be established by a carefully-considered diagnosis of each of these two diseases. This was the chief task which the author imposed on himself, when he wrote his first work, entitled the "History of Typhoid and of Typhus Fever." In the present expanded volume he begins with that of the first-mentioned disease, the symptoms, anatomical lesions, causes, varieties and forms, duration, march, and complications of which, together with its mortality and prognosis, diagnosis, theory, treatment, definition, and bibliography, he details in twelve chapters, which constitute the first part of the entire work. The same course is pursued in reference to typhus fever, in the like number of chapters, which make up Part II.

In the chapter on the diagnosis of typhus fever, we read: "In their mode of access, typhoid and typhus fevers in many instances very nearly resemble each other." The author adds, in the next paragraph, "I am not aware that there is anything in the chills or in the character of the pulse to distinguish the two diseases." Again, "There is a pretty close correspondence in the number, the severity, and the constancy of the nervous symptoms in the two diseases." A difference in this respect is pointed out by our being told, "The nervous symptoms in typhoid fever almost always creep on more stealthily and gradually than they do in typhus. This is especially true of the dulness and stupor." We are entitled to lay the more stress on the fact of the general sameness in the character of the nervous symptoms, when we reflect that the name of typhus is derived from the powerful stunning or stupefying state which accompanies this continued fever. We must remember, also, that the symptoms which more prominently attract our attention, and impart the chief physiognomy to the two diseases, are those which indicate derangements of the nervous system, either of a primary or secondary nature. It is but right, however, that we should apprise those of our readers, who may not be in possession of the work itself, that the sentences just quoted are followed by others, in which the force of the admissions made in them are qualified by observations of a different tenor, and the indicating of some differences, to which allusion will be made hereafter. The author believes that it is hardly possible to confound typhoid fever with any other affection. He says: "Setting aside then, as I do, for the present, the true typhus fever, there is no disease more readily and positively recognized than a case of well-marked typhoid fever, of extreme or even of average severity, when observed from its commencement, and followed through its entire course." The most serious disturbances of the nervous system may be absent; and it may happen that the abdominal symptoms, the diarrhœa and tympanitic distension may be wanting, and still, the author assures us, that the diagnosis may remain in no way difficult or doubtful. In contradiction with an opinion thus positively expressed, we read, in the very next paragraph the following distinct admission: "I do not mean to say by this, that typhoid fever can always be distinguished with certainty from other diseases, even when it is watched during its whole course, and by the best observers." Louis himself, has been deceived. Lombard was equally so, both in Glasgow and Dublin, when he mistook cases of typhus for typhoid fever, and predicted with confidence that, if the patients died, the intestinal lesions usually deemed characteristic of the last-mentioned disease, would certainly be found. The examinations were made; but none of the anticipated lesions were observable. Dr. Bartlett further admits that the diagnosis of typhoid fever in children, is sometimes attended with a considerable difficulty, and he points out meningitis as the disease more than any other with which it is more likely to be confounded. Typhoid fever is also, he tells us, and the observation has been made by some of the older writers, sometimes more or less mixed up with and influenced by the pathological element of periodicity; as during the prevalence of remittent and intermittent fevers, and in the most dangerous of these, or the pernicious of European continental writers, the congestive of our own. All these circumstances must detract not a little from the ready and positive recognition of typhoid fever, so confidently enunciated just before by the author. We shall show presently, that the difficulties are still greater than those just mentioned.

First, let us advert to the symptoms received by the author, and the school of pathology to which he belongs, as those enabling us to make up the diagnosis of typhoid fever. We pass over the prodromes and the state of the nervous system, as they offer, according to his own acknowledgment, nothing materially different from what is met with in these particulars in typhus fever; although in the historical part of the diagnosis of this latter (p. 295), he attempts to indicate some points of distinction, in the mode of access and in the state of the mind, between the two diseases. The same remark applies to the state of the circulation as measured by the pulse, and the state of the heart; nor can we attach any importance to the endeavor to establish contrasts between the appearances of the tongue and incrustations on the teeth in the two fevers, for two we will call them to prevent explanatory repetitions. The abdominal organs are believed by the author to furnish less equivocal symptoms, and, accordingly, diarrhœa with thin, watery, and dark or yellowish stools, sometimes consisting of blood, "tympanitic distension of the abdomen, dulness on percussion over the spleen, and gurgling upon pressure upon the right iliac region," are enumerated with considerable emphasis, especially when compared with their alleged absence in typhus fever. Almost always, as we are told, the rose-colored "lenticular eruption will be discovered if it is timely and carefully sought for," p. 132. Farther back, under the head of "Symptoms," we read: "There is good reason to think that this eruption is almost always an invariable accompaniment of typhoid fever, in number various, and sprinkled pretty abundantly over the chest and abdomen." It is confessed, at the same time, that Louis, in thirty-six fatal cases in which the eruption was sought for, found it only in twenty-six. Epistaxis "is quite common in the course of typhoid fever."

The preceding are the symptoms on which the author, in common with all those who advocate the opinion of the distinct nature of the disease, chiefly rests his diagnosis of typhoid fever. We say chiefly, in reference to symptomatology proper, and the elements of diagnosis, which its study supplies during the existence of the disease and the life of the person laboring under it. We cannot conceive of the diagnosis of a disease being procurable from other sources, and in another period; either in that before its coming on, or in that after its termination in death. An examination of the dead body may reveal to us certain organic lesions which accompanied the disease, and which, in some instances, we are safe in believing, gave rise to certain characteristic or pathognomonic symptoms; but it is a perversion of terms to speak of these lesions, structural changes seen in the dead body, as symptoms; and if not symptoms, they cannot form a part of diagnosis. We may regard them either as causes or as effects of morbid actions, or morbid phenomena. If the former, they belong to etiology; if the latter, to morbid, called often pathological, anatomy; but in neither case do they come within the limits of diagnosis. This may be said to be an abstract theoretical restriction of diagnosis; but it is in fact the practical one, for our opinion of the distinctive features, or of the individuality of a disease, its diagnosis, must be made up from what is presented to our senses at an early stage, as a guide in our subsequent course of treatment, and without reference to the contingency of what may be discovered after death. We are often and truly told, that success in treating a disease turns mainly on a correct diagnosis; but if this last be incomplete until we have had an opportunity of examining the anatomical lesions after death, then must imperfection unavoidably attach to our treatment of those who recover under our care; and while they are praising us for our skill in rescuing them from death, we are in danger of looking at them with an air of dissatisfaction for having deprived us of the means of making our diagnosis in full.

That the author of the work now under notice takes the latitudinarian view of diagnosis to which we object, is obvious from the following passage, in which he brings forward what he deems his strongest point in support of the separate and distinct nature of typhoid fever: "I have said nothing thus far of the lesion of the elliptical plates as an element in the diagnosis of the fatal cases. It has been already remarked that this lesion is characteristic of this disease, that it is invariably found in the fatal cases of typhoid fever, and that it is not found in fatal cases of any other acute disease. If this is absolutely true, without exception and without qualification, then the presence or the absence of the lesion ought to be final and decisive in regard to the diagnosis." He, however, impliedly corrects himself in the undue stretch of signification which he gives to diagnosis, when, after going over what he believes to be the evidence in this matter, he winds up with the following declaration: "*That the connection between the diagnostic symptomatology of typhoid fever, and the entero-mesenteric lesion is, I will not say absolute and invariable, but as nearly so as the connection between the diagnostic symptoms and the characteristic lesions of any given disease whatever in the nosology in which the connection is not established by positive physical signs*,"* p. 140. We will not stop to criticise the pleonasm of "diagnostic symptomatology," or of "diagnostic symptoms," in the satisfaction we feel at finding diagnosis for the nonce reduced to its proper limits. As respects the soundness of the deduction thus formally uttered by the author, we could wish that he had postponed it until after he had introduced the leading features of the history of what he calls "a supposed epidemic typhus fever, which prevailed at Rheims, between the first of October, 1839, and April, 1840, by M. H. Landouzy,"† p. 286–9. Most of the symptoms of this local fever, for it began in and was almost exclusively confined to the prison, were those of typhus, notwithstanding which, in the six autopsies of those who had died from the disease, "the intestinal lesions characteristic of typhoid fever were present."

Before we advert to the prominent symptoms of typhus, which are supposed to distinguish it from typhoid fever, and to allow of the latter standing out with more evident individuality, we must mention, in addition to the intestinal lesions, the changes in the mesenteric glands and the spleen, which complete the series of evidence on one side, in this argument de-

* The passage is thus italicized in the work itself.

† The history of this fever is given by its author in the *Archives Générales de Médecine*, for January and February, 1842.

duced from organic alterations in the abdomen. "The glands of the mesentery are always found more or less changed, according to their position, and according to the period at which the disease has terminated." "The diseased glands correspond, very nearly, to the altered elliptical plates; those nearest the ileo-cæcal valve being more changed in their appearance," p. 84. The spleen is almost always more or less altered in appearance. The most constant change consists in the augmentation of its volume. In many cases, it is three or four times as large as it is in its natural state. It is also very generally diminished in consistence, p. 85. The lesions consisting in an alternation of the elliptical plates of the small intestine (Peyer's glands) and of the glands of the mesentery corresponding to these altered plates, make up "one of the constituent elements" of the disease, p. 87. It is the marked character and frequency, not to say constancy, of these lesions, which have led the German medical writers and pathologists to designate the fever called typhoid by the French, abdominal typhus, *typhus abdominalis*.

The symptoms of typhus fever are described by Dr. Bartlett under similar heads to those of typhoid fever. We need not follow him in his divisions and details; for, with all his painstaking on the occasion, he fails, we think, to make out his case, viz., to show that the symptoms are of such a clearly diagnostic character as to establish an essential difference between the two diseases. In his tabular summary, already adverted to, he tells us, that spontaneous diarrhœa is rare in typhus fever, and that the discharges from the bowels are not liquid; there is no gurgling on pressure over the region of the cæcum. Meteoric distension and griping pains are very rare. This is meant to contrast with the reverse state of things in typhoid fever. But in contradiction to this we learn from Huxham and Pringle, that there is rather a tendency to diarrhœa in typhus fever. The first of these writers says, that a gentle diarrhœa is often very beneficial; he gives a case, however, of violent purging, supervening on the tenth day of the disease, in which also "the belly swelled exceedingly." He mentions, as a very bad symptom, when the belly continues hard, swollen, and tender after profuse stools. Diarrhœa and tympanites were occurrences of great frequency in the histories of typhus, prison, and camp fever, collected by M. Gaultier de Claubry, in his prize memoir* on "The Analogies and Differences between Typhus and Typhoid Fever," afterwards republished with additions in a separate volume. Of all the symptoms which are supposed to distinguish the two fevers from each other, most stress would seem to be laid on the cutaneous eruptions. Those in typhoid fever being bright, scanty, and rose-colored; whereas those in typhus fever consist, in grave cases, of more abundant petechiæ, which do not disappear on pressure,

* In Mémoires de l'Académie Royale de Médecine, Tome Septième, 1838.

as the first-mentioned so readily do. In other cases of typhus fever there is no eruption. This last does not constitute even a negative point of difference, for the same remark has been made of the absence of the alleged specific eruption in typhoid fever. Louis, as previously stated, met with it in twenty-six out of thirty-six cases, or in a little more than two-thirds. Dr. Flint, quoted by the editor, Dr. Clark, found the eruption only in fifty-nine of his seventy-three cases; nearly the same proportion of two-thirds of the whole number.

We propose now to examine somewhat particularly the validity of the diagnosis of the two forms of fever, which is intended to establish a positive nosological difference between them, derived from the presence of diarrhœa and tympanites in the one, and their alleged absence in the other, and from the different appearance and other characters of the eruptions in the two diseases. The argument in favor of the separate and distinct nature of the two must fail entirely if it can be shown that, in some of the worst forms of typhus fever, in situations and among subjects calculated to impart to it an eminently malignant character, as measured by suddenness of attack, violence of symptoms, brevity of course and fatal termination, symptoms supposed to be peculiarly diagnostic of typhoid fever have nevertheless prevailed. That these phenomena do occur in a co-ordinate relation, in many cases, is proved by the testimony of most of the best historians of typhus fever. Huxham, in his account of the disease, describes a maculated eruption of different hues, black, livid, green, or chestnut, and includes among them small lenticular maculæ (*lentiginibus similes*); also white miliary pustules (sudamina?) and a red itching exanthem. Hildenbrand, in his "Treatise on the Nature, Cause, and Treatment of Contagious Typhus" (Dr. Gross's translation), is careful to distinguish the exanthem proper from petechiæ: the first is a mere red-spotted exanthematous eruption, closely resembling purpura rubra, which is very early succeeded by sudamina and by small red pustular elevations, not unlike the purple pustules that occur in malignant fevers. Do not these eruptions resemble closely the ones described in the work before us, which are alleged to be peculiar to typhoid fever? But there is yet the other form of eruption noticed by Hildenbrand in typhus, and which is commonly supposed to be among the most distinguishing symptoms of this disease. We refer to the petechiæ, which the German author says may occur with or without the red purple efflorescence, but which do not belong to the essential phenomena of typhus, and are developed only in consequence of certain requisite conditions. He truly adds, that they are not always found in this fever. Coupling this observation with another in a subsequent paragraph, that the exanthematous eruptions, which are peculiar to typhus, occur either under the form of spots or of glandulous or tuberculous specks, and again (p. 61), that the exanthematous eruption in typhus presents as many varieties and modifications as there are species, we can hardly fail to see that eruptions which are by many believed to be diagnostic of typhoid and typhus fever, appear together, or are blended in the same case. The exanthem proper, according to Hildenbrand's observation, comes out about the fourth, sometimes not till the seventh, day; the petechiæ often not till the eighth day, or even later; the first accompany the catarrhal or inflammatory stage; the second show themselves in the next or nervous stage, and often only after the disappearance of the other. *Epistaxis* is noted by Hildenbrand as generally occurring on the fourth day of the disease—a fact not justifying the remark in the "tabular summary" that epistaxis is more common in typhoid than in typhus fever. We shall find in the sequel that this assertion is farther contradicted by the testimony of other experienced observers.

Gaultier de Claubry, taking Pringle's description of typhus fever, under the names of hospital, jail, and camp fever, as used by the English author, for his standard of comparison, introduces successive accounts of the disease, recognizable by the same symptoms, which showed itself in different countries of Europe in the early part of the present century, as an attendant, or a concomitant of camps, cities in a state of siege, &c. In many, it may be said in most of these accounts, there are, however, noticeable features which by the Louis school, as represented by Dr. Bartlett, are alleged to be characteristic of typhoid fever, but which we cannot help regarding as varieties of typhus, resembling so closely as these do the universally-received picture of this fever. The family traits are unmistakable; the difference consisting only in a somewhat deeper coloring, and in parts more shading in the typhus than the typhoid portrait. We proceed to notice some of the abstracts introduced by M. de Gaultier de Claubry in his Memoir. Roux, in a description of what he terms the contagious adynamic fever which prevailed in the summer quarter of 1809, at the Josephine Hospital of Vienna, when that city was occupied by the French army, mentions, among the fully-detailed symptoms of typhus, repeated epistaxis, tympanites, diarrhœa, and the appearance of spots resembling miliary eruption in some, and reddish and purplish in other cases. Hufeland, in a violent epidemic nervous fever, which he speaks of as similar to that designated by the terms pestilential fever, putrid fever, or typhus, and which caused great havoc in Prussia in 1806 and 1807, mentions diarrhœa as precursory to the more characteristic symptoms of the disease. When these were developed, tympanites and tenderness of the abdomen, with increase of diarrhœa, manifested themselves; and also rose-colored spots, and sometimes petechiæ. MM. Brasser and Rampont describe a destructive epidemic, which, in 1805 and 1806, extended from Austerlitz to Augsburg, and which was ushered in by either a violent frontal or a sinciputal headache: the symptoms were sometimes of a catarrhal, less seldom of a gastric, and, on occasions, in young and strongly sanguineous subjects, of an inflammatory character. These were succeeded by the obvious symptoms of typhus, blended with others which would be considered diagnostic of what, in the language of the day, is called typhoid fever; such as a rose-colored eruption on the sixth day, distinct from the petechiæ which appeared later, followed by tympanites, and, in cases, by inflammation of the parotid glands. This last is mentioned by Pringle among the occurrences in typhus fever. Bourges, in his account of the diseases which he observed in the City of Bamberg, in the Grand Duchy of Warsaw, met with the hospital, the malignant putrid, the nervous, or the adynamico-ataxic fever of some authors. He specifies, among other symptoms of typhus, an acrid heat of the skin, and an aggravated condition of things when diarrhœa sets in early. M. Ducastaing (Thesis, 1815) gives a full description of a typhus fever which destroyed the greater part of those whom it attacked. The chief subjects were young conscript soldiers, who proving refractory, were, after long and fatiguing marches from home, confined in prisons in Gaieta, in the Kingdom of Naples. With the worst symptoms of typhus were associated liquid intestinal discharges, and but seldom constipation, also tenderness and enlargement of the abdomen. Petechiæ and gangrenous eschars were met with. Organic lesions were detected after death in the brain, lungs, liver, spleen, small intestines, and lymphatic glands. We shall advert to these again. Reveillé-Parise (Thesis, 1815) is the historian of the epidemic typhus fever, which committed such frightful ravages among the unfortunate inhabitants of Saragossa, towards the conclusion of the memorable siege by the French in 1809; and also in the ambulances of the troops of the latter. In addition to the symptoms indicating the most malignant form of this disease, diarrhœa occurred in nearly all the cases. Rose-colored exanthems and petechiæ were more commonly seen in the city than in the army ambulances. Parotitis was sometimes met with, and often large gangrenous eschars were formed on the parts of the body subjected to pressure. If a more rapid course and fatal termination of typhus be, as they are in the volume of Dr. Bartlett, placed in contrast with the longer duration of typhoid fever, then must the fever of Saragossa rank as pre-eminently belonging to the former category; for in some cases, called by Reveillé-Parise *typhus siderans*, death took place on the second, or, at the latest, on the third day. In the accounts of the typhus fever in the English army on the Island of Walcheren, in 1809, by M. Tresat, and among the French prisoners on board the hulks, or rather floating tombs, at Plymouth, in 1810, by M. Bouchat, we do not find the typhoid elements. A very interesting history of what its author calls the *contagious typhus*, which destroyed two-thirds of the French garrison at Dantzic, in 1813, is given by M. Tort. The disease began among the troops in barracks; and afterwards, when they were distributed among the private houses, it spread among the inhabitants. It did not always pursue a uniform march; but the picture, as drawn by M. Tort, exhibits the worst symptoms of typhus, and together with enlargement and tenderness of the abdomen, particularly of the right side. Death generally took place on the ninth day. Nasal hemorrhages showed themselves on the fourth and fifth day: there was a purple exanthem elevated above the skin, and frequently petechiæ. Sometimes parotitis occurred, and in such cases death always followed. In the memorable epidemic fever at Torgau, which carried off one-half of the garrison in 1813, we find three forms described by M. Gilles de Tourrette. The first more nearly resembles the typhoid fever of the present time, in the rose-colored spots on the skin of the abdomen and lower part of the chest on the fifth day, and nasal hemorrhage, and at a later period tympanites: the involuntary alvine discharges of a dark fetid character, come more properly, however, under the typhus head. The second form was often not ushered in by any precursory symptoms, but made its attack with great violence, and terminated fatally in three and even two days, and sometimes with the frightful suddenness of only six and twelve hours' sickness. In this form so terrifically typhus, we find tympanites enumerated, but instead of rose-colored spots, petechiæ. M. Laurent relates, in his thesis (1815), a history of the terrible epidemic typhus which committed such havoc in the French garrison and the inhabitants of Metz in 1813–14. He describes three forms: one designated by him as *typhus siderans*, from the suddenness of the attack, as if the patient were struck by lightning, and the short duration of the fever, which ended fatally often in twenty-four hours, and at the farthest on the third day. Among the symptoms were tympanites, involuntary diarrhœal discharges, and "adynamic petechiæ." The second form began with the appearances of an inflammatory fever, but soon lapsed into an evident typhus, with copious diarrhœa and epistaxis, and an eruption of red spots, and also of petechiæ. The third took the lingering course, and the features of slow nervous fever, with tender abdomen, and involuntary copious alvine discharges. M. Gaultier copies from different histories of typhus fever in France, which he tells us in advance, exhibits always and throughout an identical symptomatology. We meet in these accounts with the so-called typhoid symptoms, tympanites, diarrhœa, and rose-colored spots, allied to the most aggravated forms of typhus, and in which petechiæ are seen at the same time.

M. Gaultier, after having given these abstracts of the histories of typhus fever, as it occurred in camps, hospitals, and prisons, its universally recognized nursery and home, furnishes details of the symptoms in the successive stages of typhoid fever, drawn from the French writers, and particularly Chomel; and he arrives at the conclusion, in reference to their symptomatology, that the two diseases exhibit not only a close analogy, but the most perfect resemblance, the one to the other. This writer then compares the intensity of the two; not, however, by selecting cases of *typhus siderans*, which destroys its victims in twenty-four, or even in twelve hours, on the one side, and mild and solitary cases of typhoid fever, on the other,—although even such contrasts as these would not furnish ground for admitting essential differences, any more than are seen after comparing a case of discrete with one of confluent small-pox. Both fevers vary greatly in their degrees of intensity and resulting mortality; so that we may see mild typhus and destructive typhoid. Huxham, quoted by Dr. Bartlett in terms of approval for the description which he gives of slow nervous [typhoid] fever, as a distinct affection from typhus proper, or "the putrid, malignant, pestilential, petechial fever," expresses, it seems to us, the real nature of the difference, as being only in degree, and not in kind, by his comparing the contagion, from which the former "may sometimes arise," "to the virus of a mad dog, which works but slowly;" and the contagion of the latter to "the poison of a viper, which immediately affects and destroys the texture of the blood-globules, and brings on a very speedy corruption." Although Huxham believes the two fevers to have a very different origin, symptoms, and method of cure, he admits that "the one may be, and very often is blended with the other." This blending has been fully exhibited in the descriptions collected by the French writer, so repeatedly referred to. It is not, we must remember, merely the occurrence of epi-phenomena, or an intercurrent disease in an existing epidemic, but a true blending of symptoms derived from each, which, of course, implies a considerable sameness of character, and general harmony of details in the two to allow of such a result.

The conclusions drawn by M. Gaultier de Claubry, in favor of the sameness of typhus and typhoid fevers, are not directly met by the author of the work before us. The latter endeavors to weaken their force by altering the terms of the original thesis, and thus placing himself and the French writer as advocates of different questions. Thus we read: "In regard to the memoir of Gaultier de Claubry, it is important to state that his object is to demonstrate the identity of the typhoid fever of Paris and the jail, army, and camp fevers of the continent of Europe. *He formally puts aside the British typhus.*"* We are at a loss to find the grounds on which this last assertion is made. At the very outset, M. Gaultier tells

* This passage is placed in italics by the author.

the Academy of Medicine, in reference to the meaning which should be attached to the hospital typhus, "typhus nosocomial," that, throughout his memoir, he supposes the Academy to have had in view solely the *typhus of camps, of ships,* and *of prisons,* and, in order to prevent any misconception, he will take "as a type and point for comparison, the epidemic in the English army observed by Pringle, from 1742 to 1745; and some of those which, during the great wars of the Empire, from 1805 to 1814, caused such ravages in Germany and Spain, and on board the prison ships at Plymouth, in several provinces of France, and finally, in the hospital of Salpêtrière in Paris." In this programme our readers must see that there is neither a formal nor an implied putting aside of the British typhus by the French writer.

The American author, in continuation of the paragraph just now quoted by us, says: "He [Gaultier de Claubry] insists, as though it were one of the strongest points of his argument, upon the constant presence, in the continental jail and camp fever, of the lesions of Peyer's glands. The question has since been very fully discussed by the French Royal Academy. I only wish it to be clearly understood, that the question is quite different from the one now before us;" p. 235. It seems to us, that, if this is not the question, it is at any rate a legitimate part of the identical question, which it is the great aim of the author, through the larger division of his volume, to solve in a certain sense, viz.: by showing the essential or fundamental dissimilarity of typhus and typhoid fevers. The French writer, on the contrary, insists on the identity of the two diseases; and in proof of his position adduces a large body of evidence, chiefly derived from the accounts of typhus fever as it had prevailed, and was observed in different parts of continental Europe. Our author believes that "they are separated clearly and broadly from each other, by the presence in one and the absence from the other of very strongly-marked and constant anatomical lesions, and of groups of symptoms equally striking, constant, and characteristic;" p. 291. The French writer attempts to show that the constancy of lesions in one fever is paralleled by equal constancy in the other:—he fully succeeds in showing that the groups of symptoms in typhus include some of those which are insisted on as unequivocally diagnostic of typhoid fever. To this the American author replies, by asserting, as the result of his conviction after reading the memoir, "that many of the epidemics prevailing in the armies and prisons throughout various portions of the continent, from 1804 to 1814, corresponded in all respects to *typhoid fever,* while in other instances the disease was the Irish *typhus.*" It is to be regretted that Dr. Bartlett did not specify the epidemics belonging to the divisions, thus arbitrarily made, and show that the lesions which he deems to be characteristic of typhoid fever were absent in those which he has put under the other head. Having failed to do this, his summary way of disposing of the question by virtually taxing the French writer with ignorance of the ground he occupies in the argument, and of confounding in his description two diseases, the features of which he had made it his business to study with the greatest care, cannot meet with favor.

Dr. Bartlett's caution in weighing the value of evidence sometimes forsakes him in a natural desire to establish his favorite position; and both in his claiming and rejecting witnesses he, on these occasions, betrays, without his being conscious of it, somewhat of the eagerness of a partisan. In addition to the present instance just furnished, we may refer, in illustration of our meaning, to the strained construction which he puts on the essay on *Typhus* Fever, by Dr. Nathan Smith, of New Haven, in declaring it to be a most valuable work on typhoid fever; and, as such, he draws freely from its pages in his own account of this latter disease. That Dr. Smith did not adopt, without due deliberation and a knowledge of its nosological value, the name of typhus to designate the fever on which he wrote, will hardly be contested. If, therefore, we find him, as editor of the treatise on Febrile Diseases, by Dr. A. P. Wilson Philip—Hartford, 1816, Vols. 2—declaring that "the author's description of the typhus fever corresponds so exactly with the appearance of the disease known by that name in this country, as to leave no doubt on the mind of its being essentially the same; and he has described the symptoms so minutely that nothing need be added under this head," the conclusion is irresistible, that the continued fever of New England, with which Dr. Smith was so familiar, was "*essentially* the same" as the English typhus. But Dr. Bartlett says that Dr. Smith's typhus is the same disease as the typhoid fever; and Dr. Smith says, that his (the New England) typhus is the same as the English typhus. Now, as two things which resemble, in every particular, a third thing, must resemble each other, so, as the English typhus and the typhoid fever both resemble the continued fever of New England, known to and described by Dr. Smith, they must resemble one another and constitute an identical disease.—q. e. d. Even were it admitted that Dr. Smith was mistaken, when he felt so confident of the picture of typhus fever drawn by the English author (Philip) being a correct representation of the fever with which he was so familiar in New England, and which he called typhus, and Dr. Bartlett typhoid, can any other inference be drawn than the great difficulty of making a diagnosis which should establish an essential difference between the two? So, also, if Gaultier de Claubry, with the standard of English typhus before him, as delineated by Pringle, and a full knowledge of the alleged characteristics of typhoid fever, confounds, in his description of the camp and hospital fever of the continent, typhus and typhoid fevers, must there not be as strong a resemblance between the two diseases, as there is between two varieties of any one fever—common intermittent and congestive, mild and malignant scarlatina, discrete and confluent small-pox, for example? If such men as Smith and Gaultier, both of them close students and careful observers, are thus liable to be mistaken, is not Dr. Bartlett too sanguine in assuring us that the diagnosis of typhoid fever is easily and thoroughly made out?

Additional evidence of the difficulty of diagnosis in the two fevers, on account of their running into each other is offered in a late work by Professor Magnus Huss, of Stockholm (Sweden), on the "Statistics and Treatment of Typhus and Typhoid Fever." It contains the result of twelve years' experience (1840–1852), gained at the Seraphim Hospital, in Stockholm. The conclusion which Dr. Huss draws from observations made by himself and his colleague, Professor Malmsten, on 3186 typhus patients,—for under this head he includes both those who had suffered from typhus proper (*typhus petechialis*), and from typhoid fever (*typhus abdominalis*),—is, that these diseases, "such as they appear in the north, belong to one and the same pathological process, but that which we may call the typhus process presents several different varieties. These varieties or forms arise from the circumstance that certain groups of symptoms, sometimes from one, sometimes from another of the organs or systems, are more prominent in one individual than in another. It must certainly be allowed, that the difference may sometimes seem great enough to warrant the admission of distinct diseases instead of only varieties of the same; but, considering the intermediary form between the two, and how they often change from one to the other, and how they unite, the result must be their classification under the same pathological process. It may be very easy to distinguish the two remotest links in the chain of which the typhus process may be said to be composed, and then to decide if any particular case is one of typhus or typhoid fever, but even the most penetrating discernment may be baffled in attempting to distinguish one of the intermediary forms, which lie between the two extremes, and partake a little of each, and it may perhaps be impossible to make a correct diagnosis." —p. 5–6. This is the language of philosophic doubt uttered after extensive and prolonged observation. It is not confined to the Swedish physician. Our own Huxham,—for we cannot allow the Atlantic to cut us off from our hereditary claims on the riches of English medical lore, and the glories of English medical worthies,—expressed himself more than a century ago in nearly similar terms. When describing these two fevers (the slow nervous and the putrid malignant), he says, in words already quoted, that he is "very sensible the one may be, and very often is, blended with the other."

Dr. Huss states the grounds on which his conclusion is founded. It rests on his own observations and those of other Swedish physicians, and, although these do not in all respects agree with the observations made in other parts of Europe, they are coincident, in the main, with the statements of the majority of German writers. The facts which epidemics furnish are mentioned in the first place. We learn that in two of these, one in 1841–2, and the other in 1845–6, giving to the wards of the Seraphim Hospital, in the first period, 503, and in the second 414 fever patients, the cases were neither typhus nor typhoid fever exclusively. But, on the contrary, there were some of both, "so that in the beginning and to the height of the epidemic the cases were, for the most part, typhus, and at the end the typhoid fever almost entirely prevailed. This statement is founded not merely on the symptoms each individual case presented, but also on the results of the post-mortem examinations. With the exception of four, all who died were examined; there were 55 fatal cases in the former epidemic, 33 in the latter. Of the former 55, 36 presented those alterations of the intestinal tube and mesenteric glands which are peculiar to typhoid fever, and 19 no such alterations. Of the latter 33, only 29 were examined; of these, in 19 the glands were changed in different degrees; the remaining 10 showed no change." In a note to this passage, the author adverts to another epidemic in the Seraphim Hospital in 1852, which had every character of the exclusively abdominal form; all the *post-mortem* examinations, with the exception of one only, exhibiting the usual changes in the intestinal glands. Another epidemic, of a more limited extent, was studied by Huss, with the following results. "At the barracks of the *gens d'armes*, out of 250 men 64 were taken ill in the course of six weeks. Although the men lived under exactly the same circumstances, were exposed to the same etiological agents, were all between 20 and 40 years of age, the disease assumed the distinct forms of typhus in one part of the cases, of typhoid in another, and in a third took an intermediate form. In the private house of a cabinet maker I saw 17 cases within a fortnight, of which 10 were typhus and 7 typhoid fever, although here also the dwelling and other circumstances, with the exception of age and sex, were the same for all." Two cases are mentioned of a man and his wife contracting fever by their living in the house and using the clothes of his brother, who it was stated had died of typhus. "They were soon taken ill and brought to the hospital, where they both died. The husband had violent delirium and a profuse petechial eruption, the *post-mortem* examination showing no change of the intestinal glands; the wife had milder cerebral symptoms, and a very scarce crop of eruption; but on examination swollen mesenteric glands and swollen and ulcerated Peyer plagues were found in abundance." In the epidemic typhus cases, noticed by the author, "a number of intermediate cases were of very frequent occurrence, making it utterly impossible to determine the true nature of the case, whether petechial or abdominal, as there were symptoms of both. Often such symptoms were observed as seemed to announce an affection of the intestinal glands, but the *post-mortem* examination did not discover any; in other cases these alterations were found, and the symptoms had indicated the petechial form."

Reference is made by Huss to the striking differences in the violence of the symptoms and the number and extent of organic lesions in the varieties of scarlet fever. Some fifty patients of both sexes, between 51 and 25 years of age, laboring under this disease, were entered at the Seraphim Hospital. "In these cases there was, with the exception of the cutaneous eruption, a greater variety in the symptoms presented than is ever found in those of the typhus forms." After describing two cases of scarlet fever in which the author saw appearances so frequently found in petechial typhus; and in another case, which had begun with the intestinal symptoms, the glands of Peyer were affected in the like manner as in typhus abdominalis. The difference between these forms of scarlet fever appears to him to be quite as great as that between typhus petechialis and typhus abdominalis; and, he continues, when, notwithstanding this, the different forms of scarlet fever are brought together as one disease, it seems to him but right to adopt an analogous junction in reference to the typhus fevers. The chief objection to establishing this analogy is, in his opinion, the uniform and decided character of the eruption in every form of scarlet fever, in opposition to the diverse cutaneous eruptions admitted to characterize the different forms of typhus. The first part of this observation, or that relating to scarlet fever, is laid down too broadly. We have seen, in the present epidemic of this fever, and our experience is doubtless not solitary, cases of peculiar malignancy, and which terminated fatally, without there having been any eruption at all.

Huss examines some of the symptoms and anatomico-pathological lesions, which are especially considered to mark the distinction between typhus petechialis, and typhus abdominalis or typhoid fever. He passes in review the eruptions on the skin, the cerebral and nervous symptoms, and the state of the organs of circulation, without his being able to find the distinctive differences sought for. More promise of such a result is offered in the state of the organs of digestion, and especially of the intestinal canal; but even here, while admitting the very frequent occurrence of the symptoms in typhus abdominalis, which French authors look upon as diagnostic, the author assures us that not only may a single one be wanting, but all of them at the same time. He next inquires into the results reached by dissection, and takes occasion to rebuke mildly those persons who, notwithstanding all the symptoms are distinctly marked, refuse to see a case which offers them as one of typhoid fever, unless the ordinary lesions be found; or, on the other hand, who will insist on a case being one of typhoid fever, if ulceration of the glands of Peyer is present, notwithstanding all the symptoms had been characteristic of petechial typhus. He thinks it must be allowed that typhoid fever can exist without affected intestinal glands, unless we suppose those physicians who have communicated these statements incapable of making a correct diagnosis. The other anatomical lesions afford, he thinks, no marks of distinction. The symptoms in the two diseases exhibit a difference in degree of intensity, but scarcely anything more. The attempt to draw a line of distinction between the two forms, typhus and typhoid, furnished by etiology, has not been successful. Both are liable to be propagated by contagion, but typhus in a greater degree, although the relative contagiousness of the two diseases is much influenced by the varied nature of different epidemics. The more important question, whether the contagion which produces typhus differs materially from that causing typhoid fever, is replied to in the negative by Huss, who believes it is difficult to deny that the same contagion can produce the two forms. But he admits that it is difficult, if not altogether impossible, to find out the individual circumstances on which the disease seems to depend. He believes constitution and temperament to have a decided causative influence. The limits in regard to the age of the patient, within which it was at first taught that typhoid fever was restricted, have since been considerably extended; but if what has been said on this subject be ever so true, it does not furnish, in the opinion of the author, a sufficient reason for placing typhus and typhoid as two different morbid processes. In some other points of etiology, especially in their sporadic origin, "the two maladies are perfectly congruous; unhealthy, overcrowded, ill-ventilated dwellings, poverty and its privations, care and sorrow, and a certain process of acclimation for such persons as come from the country to inhabit large cities, &c. &c., being the remote causes of both." The Swedish author sums up his creed in the following declaration, which we should be willing to utter as our own, viz.: "That typhus and typhoid fever (typhus petechialis et typhus abdominalis), such as they appear in the climate of the North, neither can nor ought to be considered as two distinct maladies, but only as two varieties of the same morbid process." This, though a decided, is a less extreme view of the question than that taken by Gaultier de Claubry, who in insisting on absolute identity claims too much, and especially on the point of intestinal lesions, which although met with in some of the epidemics described by him, have not been proved to be present in all, nor do we believe that such constancy of appearance exists in the continental typhus: we know that it does not in the British Islands.

The frequent use of the additive *abdominalis*, by the Swedish writer, who follows the German nosographists, to designate that variety of typhus which, in France, England, and the United States, is generally termed typhoid, must have been noticed by our readers in previous extracts from Dr. Huss's volume. Conformably with the pathological views which he, and others who think like him, entertain on the subject, either of these names might be considered appropriate; the first as indicating a variety of typhus in which some of the leading symptoms and the most important organic lesions are abdominal; the second, or typhoid, as conveying the idea of a modification of or resemblance to typhus. In this sense we speak of typhoid pneumonia, inflammatory fever resulting from local injury running into a typhoid state, erysipelas of a typhoid character, just as varioloid is used to represent an eruptive fever resembling or having symptoms similar to variola or small-pox; or cinchonoid, a quality or qualities belonging to cinchona, or asteroid as a minor star, or luminous body resembling a star. We see in all these cases abstract names, qualities or attributes of an antecedent concrete name. Typhus is the concrete, typhoid the abstract name. To say of typhoid that it can stand for or be the expletive of a thing or state of things entirely dissimilar and different from typhus, is to predicate of a quality or attribute entire unlikeness to the substance or thing of which it is a quality or an attribute. It is an abuse of words, a perversion of all the analogies of our language, to call a fever which is essentially different from typhus by the term typhoid: and so long as the designation is attempted by those who hold to this pathology, so long will they find themselves continually embarrassed in establishing their position, not only by the difficulty of the thing itself, but by the confusion created by their wrong terminology. If they wish to show that the fever in question has a fixed organic connection, they cannot say origin, let them call it with the Germans *typhus abdominalis;* or if they believe that its somewhat longer and slower progress are characteristic, and that it affects strongly the nervous system, let them call it after Huxham, the *slow nervous fever.* If we could name a fever after an organ or organs which are most generally the seat of anatomical lesions, the most appropriate designation would be *entero-mesenteric* for the fever in question, as proposed by Petit and Serres. The term enteric is adopted by Dr. Dickson, who follows in this respect Dr. Wood. But in the present state of the pathology of fevers we are not prepared to name or classify them from their supposed seat in any one viscus. No visceral inflammation, separated from other pathological causes or influences, can light up a fever which shall represent fully, still less be identical with, any of those now regarded as idiopathic.

Dr. Dickson, in adverting to the subject of the alleged difference between typhus and typhoid fever, believes that "the cases arrange themselves in groups, resembling each other generally in the prominent symptoms, and requiring similar management." He subdivides typhus fever into four varieties, viz.: 1. The simple typhus of Armstrong, mild typhus, the nervous fever of common phraseology; 2. Abdominal typhus, typhoid or typhoidal fever, typhoid affection, dothinenteritis, ileo-colitis, enteric fever; 3. Cerebral typhus, in which coma or stupor is the prominent feature, the tendency to which indeed gave name to the whole class; 4. Putrid fever, malignant typhus, jail, ship, and hospital fever. The first two come fairly under the head of typhus mitior, which will include the lighter cases also of the third; the fourth is the typhus gravior of Cullen, and the older English writers, and will comprise some of the severer instances of the third.

The Carolina Professor adverts to the doctrine held by Rokitansky, of the generation of a typhus poison in the blood—typhosis, which must be got rid of or thrown off; and its elimination is through the intestines, whence the peculiar deposit and consequent pustulation. The analysis of the typhus matter makes it very nearly identical with tuberculosis and scrofulosis.

Little space is left for us to speak of the treatment of the two forms of fever, typhoid and typhus, contained in Dr. Bartlett's volume. The author has not introduced anything new or suggestive, nor has he made any attempt to transfer the treatment of these diseases from the empirical ground on which they now rest to one of principles. Perhaps a needless exhibition of our therapeutic weakness is made by him, in giving under the several heads, Dr. Jackson's method, Dr. Nathan Smith's method, Chomel's method, &c. &c., to which the editor has added Huss's treatment of typhoid fever. The most noticeable feature in this last is the use of compresses wet with cold water, applied to the whole abdomen. Huss believes that this application, preceded by sinapisms or fomentations with turpentine, and continued daily till the approach of convalescence, has great influence in diminishing the abdominal lesions and symptoms. We have used the abdominal compress and the wet sheet in two different cases of typhus fever, with obviously good effects. The lives of the patients seemed to be saved by the remedy. Dr. Bartlett enumerates the remedies in typhus fever in the same way as he does the symptoms, and with a similar disadvantage, of isolation, when we wish to know the effects of remedies given in different stages of the disease, and of their alternate or combined use. A good outline of treatment, both of typhoid and typhus fevers, in this last spirit, is given by Dr. Dickson.

We cannot follow the author in his history of periodical fever, the several parts of which are well written, and present a clearly-drawn but not a full picture of the varieties usually recognized. He has restricted himself for the most part to French and American writers, to a neglect of some excellent

English and Italian ones. Among the latter he dismisses the great names of Torti, Baglivi, Lancisi, and Rammazzini, with the compliment of calling them classic authorities upon the subject, and the additional remark: "But their descriptions are generally inaccessible here, and they are of course but little known; it should be said further that they are quite deficient in pathological details." The narrow and incorrect meaning given by so many of the modern writers to the term pathological, restricting it to designate morbid anatomy, instead of all that relates to the morbid condition of the body during disease, makes the author do injustice to these Italian celebrities. The fact mentioned in the above extract, of their being little known and generally inaccessible, seems to us to be a reason for their peculiar merits being brought out by a historian of periodical fever. In another page, he speaks of them not being at hand. If they had been perused by the author, he would not have introduced the four names without any qualifying remark, as if they had all written with equal fulness and instruction on the subject of periodical fever. Baglivi, snatched away in the prime of life, from the field of medical science, of which he promised to be an original and successful, as he was a brilliant cultivator, speaks of the semitertian or hemitritæa and the kindred tritaeophya, as the common autumnal fevers of Rome, and as both violent in their progress and often fatal in their termination. The organ which evinces the greatest derangement and distress is the stomach. Sometimes the fever assumes an algid, sometimes a comatose, or, as he calls it, apoplectic form. In sections *De Febribus in Genere*, and *De Febribus Malignis et Mesentericis*, —*Opera Omnia*. Venetiis, 1754. The work was first published in Rome, 1696. Rammazzini (*Opera Omnia—Medica et Physiologica.—Genevæ*, 1717), when detailing the epidemic constitution of Mantua and the adjoining country for 1690, speaks of the great prevalence of intermittent fevers during that year, and the severity and extreme danger from night paroxysms of double tertian. In an epistolary dissertation addressed to his nephew, on the abuse of Peruvian bark, he describes fatal cases of algid and comatose fever of the tertian type, but hesitates to administer this medicine with the requisite freedom; although prejudiced against the bark, he admits that it might be of use in these malignant intermittents, if a proper diagnosis be early attained. Lancisi (*Opera Omnia. Genevæ*, 1718), in his famous treatise on the noxious effluvia from marshes (*De Noxiis Paludum Effluviis*), describes epidemic intermittent, and its pernicious varieties, and counsels the free use of bark in doses of two scruples to a drachm, frequently repeated, for their cure. But of all the early Italian medical writers, Torti is most entitled to our regard for his full descriptions of periodical fever, and particularly of the most dangerous and fatal variety, designated by him and the continental writers as pernicious, and known to us as congestive; and also for his recommendation of large doses of bark in these cases. He gave half an ounce at a time, without particular regard to the stage of the fever, or its complication with other diseases. He comments on, and criticises with much force and point, the letter of Rammazzini just referred to. Torti wrote in 1712, and if his work, *Therapeutice Specialis, ad Febres Periodicas Perniciosas,* had been put into an English dress at the time, there would have been an incalculable saving of life in all the English possessions in tropical regions, and in our own country. A little more book-knowledge, which is supposed by non-reading routinists to be opposed to practical tact, would have been, in this case at least, a most important help in every-day practice in those extensive regions in which periodical fever in its various forms is endemic. Torti wrote four letters in Italian to Muratori, in the fourth of which he asserts his claim to be the first to show in Italy, the true nature of pernicious periodical fevers, as he was also the first beyond question to prove the certain means of curing them by the administration of large doses of Peruvian bark. He does justice to Morton, whose writings on the subject were somewhat anterior to his own. Torti draws freely in his work from Mercatus, who described well the malignant intermittent, but who was ignorant of the remedy. Morton, who is quite overlooked by our author, described some of the worst varieties of pernicious fevers, and inculcated the free use of bark for their cure (*Opera Medica. Tomus Secundus,* Cap. IX, *De Proteiforme Intermittente Febris Genio*). The free use of bark in remittent fever, even in the paroxysm, was practised with success by Dr. John Clark (*Diseases of Long Voyages*) nearly a century ago. The good service of showing the relations of European medical literature to the wants of American practice, so far as regards a better knowledge of the pathology and treatment of congestive fever was, we believe, first performed by Dr. Bell, in his written lectures added to those of Dr. Stokes. The want of recognition of the fact, that the pernicious intermittent and remittent fevers of Southern Europe were the same as our congestive fevers, deprived many of the advantages which they might have derived from the perusal and study of works which had been translated and were accessible; such as those of Senac and Alibert. A more careful study of the English writers, from Morton to Clark, would of itself have saved much guessing and groping before the right path was finally found.

The prominent points of treatment of periodical fever in its different forms of intermittent, remittent, and congestive, are set forth by Dr. Bartlett; but we miss a minute appreciation of the effects of certain remedies, as modified or rendered more efficient by others, given antecedently, or in combination with them. Thus, for example, we have repeatedly cured, or at least prevented for a long period, the recurrence of the paroxysm of intermittent fever by a single venesection, followed by quinine, which last, and other analogous remedies, had of themselves failed entirely to arrest the disease. A similar remark will apply to the timely use of calomel or blue pill, preceding that of quinine, or sometimes in alternation with it. Dr. Dickson may be consulted with much benefit on the whole subject of the pathology and treatment of the varieties of periodical fever. It is one of which he has a full knowledge in all its bearings, the result of actual observation and long experience. Every practitioner who has seen and is at all familiar with the course of bilious remittent fever must be greatly disappointed at the extremely meagre and unsatisfactory account of its treatment, contained in the three pages devoted to the subject by Dr. Bartlett. The inexperienced will find scarcely anything to guide them in the resort to suitable means for meeting the successive and alternating symptoms and changes which are met with in the sick-room on such occasions.

In turning from the outlines in this volume, which partakes more of the closet than the clinical ward, to the directions given by Dr. Dickson, the difference is very perceptible. The latter writer lays down the indications for the use of the lancet, with a clearness and precision which we miss in the former. On the use of mercury, with a view to its constitutional action, of the cold bath by immersion, affusion, and ablution, and of the vapor bath, antimonials, blisters, and diffusible stimulants, in the treatment of this fever, Dr. Bartlett is entirely silent. On each of these points Dr. Dickson touches with brevity, but with distinctness, while designating the particular condition of the system to which the remedy is deemed most appropriate. The treatment during convalescence and that for the sequelæ of the fever are also important topics, which are noticed by the Southern teacher, although overlooked by the Northern one. The former makes brief but pertinent notice of the "Country Fever," as it is called in the cities of the South. It is "a modification of bilious or malarious fever, originating in *transient* exposure to the intensely concentrated malaria of the low country, as by sleeping a night or two upon a rice-plantation during the summer and autumnal months." The prognosis is unfavorable. "The treatment must be prompt and decided. Our best dependence, from the very commencement, is upon the combination of the mercurial with sulphate of quinine in efficient doses. Ten grains of each may be given at first, and half the quantity at intervals of two hours until the bowels are moved, when the calomel may be omitted, persisting with the quinine until quininism has been induced, which must be kept up for 72 hours at least, for fear of a return of the attack, the proclivity to such relapse being very great."

Part Fourth of Dr. Bartlett's volume is taken up with the history of Yellow Fever. With the drawback already pointed out, which meets us in his accounts of the other fevers, viz., the want of a full picture of the disease and of the changes which it undergoes in its successive stages, this is the best prepared and most instructive portion of his literary labor. He has drawn the better part of his materials from American sources, and exhibits them with method and clearness. He appears to greater advantage on this occasion from his being more of the historian and less of the controversialist than in parts first and second, in which he discussed and argued the true nature of the relations of typhoid and typhus fever to each other. It would be easy for those who are in habits of yearly intercourse, we might say, with yellow fever to point out omissions; but these will not prevent a ready acknowledgment of the obvious merits of the present history. The editor, ever sympathizing with his author, has been equally happy in the appositeness and point of the additions which he has made, and which embrace views and details derived from the most recent writers on the subject. Among these we may mention, as the most prominent, Drs. Drake, La Roche, Barton, Blair; and numerous contributors to the Southern Medical Journals and to the "American Journal of the Medical Sciences," among whom we meet with the authoritative names of Dickson, Porcher, Hume, Fenner, Dowler, and Porter. Having already exceeded the limits within which we designed to restrict ourselves in our running commentaries on the works whose titles are placed at the head of this article, we cannot quote from, still less can we offer an analysis and a criticism of the views advanced respecting the many debated points on the pathology and treatment, and more especially on the etiology and prophylaxis of yellow fever. We may, perhaps, soon be called on to say something on this last theme.

